# Understanding Global HIV Stigma and Discrimination: Are Contextual Factors Sufficiently Studied? (GAP_RESEARCH_)

**DOI:** 10.3390/ijerph16111899

**Published:** 2019-05-29

**Authors:** Bach Xuan Tran, Hai Thanh Phan, Carl A. Latkin, Huong Lan Thi Nguyen, Chi Linh Hoang, Cyrus S.H. Ho, Roger C.M. Ho

**Affiliations:** 1 Institute for Preventive Medicine and Public Health, Hanoi Medical University, Hanoi 100000, Vietnam; 2 Bloomberg School of Public Health, Johns Hopkins University, Baltimore, MD 21206, USA; carl.latkin@jhu.edu; 3 Institute for Global Health Innovations, Duy Tan University, Da Nang 550000, Vietnam; haipt.ighi@gmail.com (H.T.P.); huong.ighi@gmail.com (H.L.T.N.); 4 Center of Excellence in Behavioral Medicine, Nguyen Tat Thanh University, Ho Chi Minh City, Vietnam 700000, Vietnam; chi.coentt@gmail.com (C.L.H.); pcmrhcm@nus.edu.sg (R.C.M.H.); 5 Department of Psychological Medicine, National University Hospital, Singapore 117599, Singapore; cyrushosh@gmail.com; 6 Department of Psychological Medicine, Yong Loo Lin School of Medicine, National University of Singapore, Singapore 117599, Singapore; 7 Institute for Health Innovation and Technology (iHealthtech) National University of Singapore, Singapore 117599, Singapore

**Keywords:** scientometrics, HIV, stigma, discrimination, contextual factors

## Abstract

Stigma and discrimination are among the greatest challenges that people living with human immunodeficiency virus (HIV) face, and both are known to negatively affect quality of life as well as treatment outcomes. We analyzed the growing research and current understanding of HIV-related stigma and contextual factors in HIV/AIDS (human Immunodeficiency virus/ acquired immunodeficiency syndrome) bibliography. A total of 5984 publications published from 1991 to 2017 were retrieved from the Web of Science database. The number of papers and their impacts have been considerably grown in recent years. Research landscapes related to stigma and discrimination include clinical, physical and mental health outcomes, risk behaviors of most-at-risk populations, and HIV-related services. We found a lack of empirical studies not only on social, cultural and economic contexts, but also on specific interventions for particular settings and sub-populations. This study highlights certain gaps and provides a basis for future studies and interventions on this critical issue given the changing drivers of HIV epidemics.

## 1. Introduction

According by WHO, the human immunodeficiency virus (HIV) is the virus that can destroy or impair immune system function and lead to acquired immunodeficiency syndrome (AIDS) which is known as the most advanced stages of HIV infection [1]. By the end of 2017, there were 36.9 million people living with HIV worldwide, with 1.8 million new infection cases and 940,000 deaths [2]. Therefore, HIV/AIDS is considered as a global health burden [3]. The ongoing HIV epidemic requires continued and novel strategies in order to address the biomedical and psychosocial nature of the disease [4,5].

Since its identification nearly 40 years ago [6,7], one of the toughest challenges faced by people living with HIV (PLWH) is social stigma and discrimination [8]. According to the sociologist Erving Goffman, stigma can be defined as “an attribute that links a person to an undesirable stereotype, leading other people to reduce the bearer from a whole and usual person to a tainted, discounted one” [9]. Stigma and discrimination are not only fuel the spread of epidemics, but also impact patients in their responses and living with HIV/AIDS by social isolation, stress and emotional coping, and denial of social and economic resources [5,10]. Despite much intensive understandings of HIV and advancements in HIV treatment, studies have recorded HIV stigma and discrimination within interpersonal, community, and healthcare environments. Discriminatory attitudes, HIV testing without consent, refusal of care and treatment, and confidentiality violation have been observed in a large number of healthcare workers [11,12,13]. A study by Wagner also reported high levels of discrimination and even overlapping stigmas held by health care providers in such a developed country as Canada [14].

Stigma and discrimination have affected PLWH’s life in many ways, and most of them are detrimental. HIV-related stigma is often associated with psychological distress, such as shame [15], depression [16], anxiety [17,18], suicidal ideation [19] and quality of life [20,21,22]. Furthermore, stigma towards PLWH in healthcare settings is considered one of the major barriers to optimal treatment. Numerous studies suggest that experiences of HIV-related stigma resulted in lower access to HIV treatment, low utilization of HIV care services, poorer antiretroviral therapy (ART) adherence, and thus poorer treatment outcomes [12,13,23].

Although a growing body of literature has expressed the interest in this topic, most research has focused on general issues related to HIV stigma. Marshall et al. measured the HIV-related discrimination among healthcare providers [13]. A systematic review conducted by Rueda et al. examined the associations between HIV-related stigma and health outcomes in PLWH [24]. These and other reviews provided a summary of research in this field, however, did not fully describe the trends in research and intervention designs as well as the emergence of research approaches over time. Quantifying the growth in studies and usage, which is defined as the total downloads, as well as characterizing research domains are beneficial for informing priority settings and programming, especially in a particular context. This study aims to measure the international growth in specific research interests by evaluating the tendency of research topics of published articles over time and establishing a network of research collaborations based on published literature. We also delineated the development of research landscapes for factors associated with HIV stigma and discrimination among PLWH.

## 2. Materials and Methods

### 2.1. Search Strategy

A cross-sectional study for HIV/AIDS bibliography analysis was designed based on the Web of Science database. The search query of keywords consisted of HIV; human-immunodeficiency-virus, AIDS, and acquired-immune-deficiency-syndrome. We selected two types of research paper, namely research articles and research reviews, and excluded other document types (e.g., books, book chapters or data papers). The language and publication date were also restricted. The publications in English until 2017 were chosen because this study was performed in the middle of 2018. Thus, the data produced in the first half of the year may not fully reflect the trend of the entire year. The second step was to select papers mentioning stigma and discrimination in the Abstracts or Titles.

### 2.2. Data Extraction

**Figure 1:**
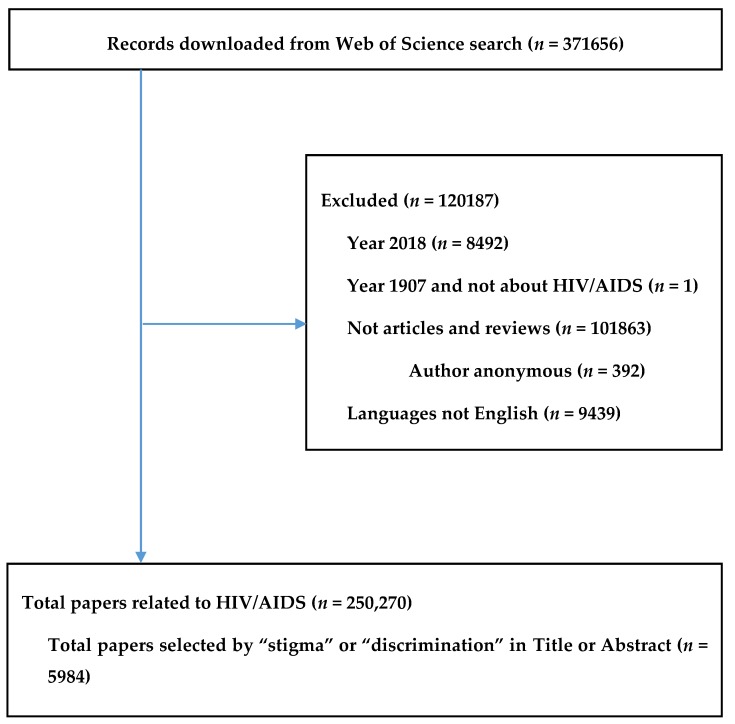
Selection of papers.

Data, including authors’ names, the papers’ titles, the journals’ names, keywords, institutional affiliations, the frequency of citation, subject categories, and abstracts, were downloaded from the Web of Science database. In addition, the citation reports automatically created by the Web of Science were also downloaded. All data were converted to xlsm form (Microsoft Excel) for data checking of errors. In order to bring together the different names of an author, a process of standardization was carried out by two researchers (Figure 1).

### 2.3. Data Analysis

We analyzed data based on general characteristics (publication years, main categories, number of authors), keywords (co-occurrence keywords and most common keywords), citations, usages, and abstracts. After downloading and extracted data, we applied Macro, a programming code run on Excel, to calculate countries’ citation, intra- and inter-country collaboration. The connection among countries sharing the co-authorships was illustrated by a network graph. The author keyword co-occurrence network and countries network were also created by VOSviewer (version 1.6.8, Center for Science and Technology, Leiden University, Leiden, the Netherlands). We applied exploratory factor analysis to identify research domains emerging from all contents of the abstracts; loadings of 0.4. Jaccard’s similarity index was utilized to identify research topics or terms most frequently co-occurring with each other. It was defined as the size of the intersection divided by the size of the union of two sets of co-occurring terms. We created illustrations of the proximity values computed on all analyzed keywords using multidimensional scaling. In the map, a point represented a topic category, and the distances between pairs of items revealed to what extent those items appeared together. Colors represented the membership of specific items to different partitions created using hierarchical clustering. Stata software was used for statistical analyses. Analysis of each type of text data are presented as follows (Table 1):

## 3. Results

### 3.1. Number of Published Items and Publication Trend

The paper selection process is displayed in Figure 1. Among 250,270 articles in 153 HIV related research areas, 5984 papers including the terms “stigma” or “discrimination” in the title or abstract were selected and analyzed.

The number of papers counted by study settings of 125 countries is presented in Table 2. The American population appears to be the most well-studied one with 406 cases, accounting for 14.26% of the total sample.

The increasing number of papers on discrimination towards people living with HIV/AIDS can be observed from Table 3. Throughout the last decade, the number of papers has been considerably grown and reached 633 articles in 2017, tripling that of 2007 and making up over 15% of total HIV/AIDS studies. Notably, the total usage of the last five years of 2003 is about 3–4 times higher compared to the previous years. Total citations and usage rates have also risen remarkably, especially from 2003. Particularly, in terms of mean use rate of the last five years, the figures for the years 2003 and 2013 were the largest throughout the research period.

### 3.2. Authors and Collaborations

**Figure 2:**
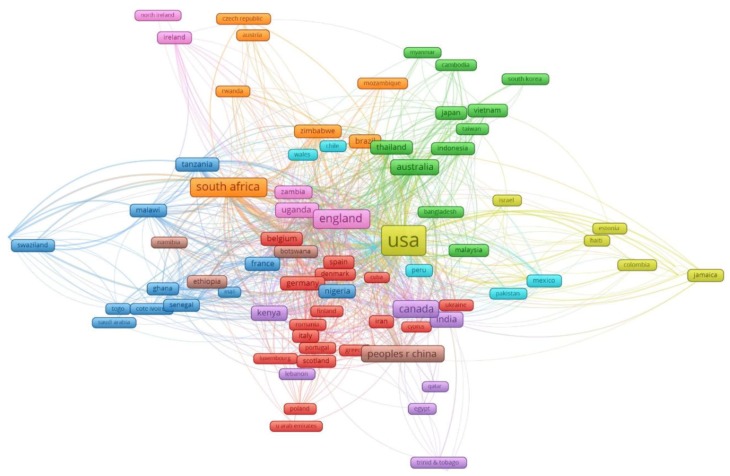
The global networking of 97 countries having co-authorships in stigma research in HIV/AIDS.

The global network among 97 countries having co-authorship of selected papers is illustrated in Figure 2. The figure was constructed by counting the co-occurrence of different countries in the authors’ affiliations, and clustering them into groups of countries with similar level of co-authorship with other countries. The size of nodes is proportional to the contribution to the number of papers and the thickness of lines represents the percentage of the number of collaboration. These countries have been classified into 9 clusters of at least five countries depending on their level of international collaborations. Each color indicates a level of international co-authorship. Apart from leading nations in global HIV initiatives, such as the United States or the United Kingdom, regional neighbors or countries sharing the same languages tend to have similar levels of international co-authorship.

### 3.3. Keywords and Research Domains

**Figure 3:**
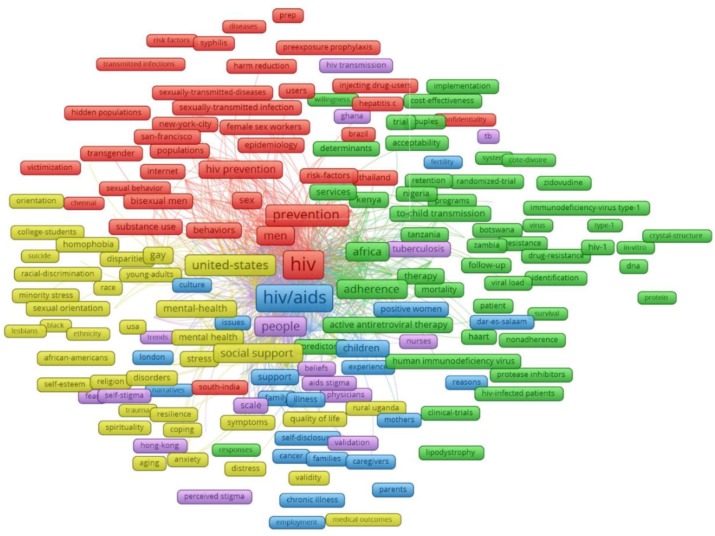
Co-occurrence of most frequent author’s keywords. Note: the colors of the nodes refer to principal components of the data structure; the nodes size was scaled to the keywords’ occurrences; the thickness of the lines was drawn based on the strength of the association between two keywords.

Figure 3 reveals the principal components of the keywords structure with the most frequent groups of terms. There were five major clusters emerging from 406 most frequent keywords co-occurrence of 20 times and higher. Cluster 1 (red) refers to prevention of HIV transmissions through sex or blood in particular subjects, including transgender, bisexual men, female sex workers and injecting drug users, in America, India, and Thailand. Cluster 2 (green) focuses on HIV medication adherence and associated matters in several African countries. Cluster 3 (yellow) reveals social issues relating to the mental health of young adults and homosexuals in the United States. Cluster 4 (blue) describes experiences of families having members with HIV-related chronic illnesses, while cluster 5 (purple) mentions two types of HIV/AIDS stigma, which are self-stigma and perceived stigma.

The top 50 research domains and keywords emerged from the exploratory factor analysis of all abstracts’ contents are listed in Table 4. Men who have sex with men, care providers, and female sex workers were the top three domains attracting greatest interest and each accounted for nearly half of total cases. Stigma and discrimination relating research domains focused on risk behaviors, clinical and quality of life outcomes, and HIV-related services uptake. Contextual factors were only found in domain # 32, which was cultural beliefs.

**Figure 4:**
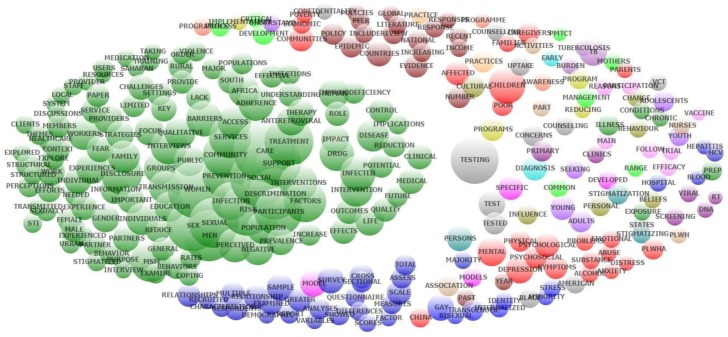
Co-occurrence of most frequent topics emerged from exploratory factor analysis of abstracts contents.

Exploratory factor analysis of abstracts’ contents also emerged co-occurrence of most frequent topics (Figure 4). A wide range of topics, such as outcomes of interventions, treatment and prevention, the role of social support and services, sexual transmission, and impact of discrimination and stigma, are specified in green nodes. Red nodes mention physical, psychological and social problems of people living with HIV/AIDS in general, whereas cluster in cobalt blue focuses on cross-sectional studies of the LGBT population. Brown cluster reveals the literature review of national policies and evidence of this epidemic. The remaining scatters on the right of the figure includes various issues. HIV testing and counseling, stigmatization of people living with HIV, vaccination and HIV-associated chronic diseases, for example, are the major concerns.

Table 5 presents the most cited papers discussing interventions and trials related to stigma and HIV/AIDS. It depicts that the interests and preferences of the research community to approach stigma and discrimination include two main areas: (1) contextual factors including social, cultural, psycho-behaviors, gender, family and community and (2) the implementation of HIV vaccine trial.

## 4. Discussion

This study reveals that the quantity and impacts, which are indicated by citations and usages, of publications on HIV-related stigma and discrimination have grown considerably, especially in recent years. Countries with large HIV populations, such as the United States of America, South Africa, China, India, and Uganda, possessed the highest number of empirical studies. Research landscapes related to stigma and discrimination include clinical, physical and mental health outcomes, risk behaviors of most-at-risk populations, and HIV-related services. We found a lack of empirical studies not only on social, cultural, and economic contexts but also on the design of specific interventions for particular contextual factors.

Findings from this study were in line with previous reviews in terms of factors associated with HIV-related stigma and discrimination. Previously, Sweileh published a review of 2509 papers relating to stigma and HIV/AIDS based on data obtained from Scopus database [25]. Nevertheless, the prior review simply focused on the bibliometric analysis without intensively examining research contents and scopes of the papers involved. Although similar trends in research growth and other bibliometric features were found in this research, since we quantified the factors associated with stigma and discrimination against HIV/AIDS patients and classified these factors into differential clusters, this study has enriched the understanding of current empirical research about stigma and discrimination and may help guide future studies. Specifically, media communication should expand the reach of existing interventions against the stigma of people living with HIV/AIDS. In addition, for designing interventions to reduce stigma, important context- and culture-specific factors should be incorporated into the characterization of causes of stigma. By precisely quantifying the frequency of research growth, we have incorporated a system-thinking perspective into the insights into HIV-related stigma and discrimination and have identified gaps, especially, about contextual factors, in the literature.

This study has several implications for improving HIV prevention, care, and treatment. It highlights a strong correlation between stigma and most at-risk populations, such as drug users, men who have sex with men and sex workers. In addition, as it is critical to eradicate stigma and discrimination in order to improve the access and utilization of HIV-related services [24,26], research on HIV-related stigma should focus more on characterizing contexts of the populations of interest, along with combining different approaches and intervention packages. An intervention, for instance, may incorporate with community-based and mass media communication to elevate its value and reduce discrimination within the population, hence, improve multiple outcomes such as mental health status, substance use, social isolation, and employment. Also, stigma and anticipated discrimination may affect patients’ quality of life due to a number of features, including family context, neighborhood, and occupation. Particularly, social judgment and stigma are highly expressed in many developing countries, where HIV epidemics mainly exist in groups exhibiting specific behaviors, such as injecting drugs and being sex workers [26]. Addressing HIV-related stigma, therefore, requires concrete empirical evidence in different contexts, which might characterize its associated factors and indicate potential effects of the interventions. Also emerged from these analyses was the association of health care services and stigma. In many circumstances, patients disclose to and seek help from health workers regarding their risk behaviors and health status. Thus, sustaining stigma reduction counseling and integrating psycho-behavioral interventions into services for PLWH may make a significant contribution to the increase of service uses and reduction of stigma against HIV-positive individuals.

Although we introduced a novel approach in summarizing and analyzing the literature, some limitations should be acknowledged. First, only publications in English were selected for this study. Another limitation is that the involved databases were limited only to the Web of Science. Despite the fact that Web of Science contains a large proportion of the literature of HIV/AIDS research, it is probably not fully representative of all data. Using the WoS database for this analysis was, however, limited in its capacity to construct indicators to describe research development. For example, we could only use the total number of downloads and the mean rates of downloads for assessing the usage. Besides, the content analysis solely consisted of abstracts instead of full texts; and the construction of different types of articles was not fully addressed in this approach of analysis. Traditionally, bibliometric analysis only focuses on number of papers and keywords instead of involving screening of abstracts because it works with a large number of papers. We enhanced the validity of such analysis by incorporating content analysis and scrutiny of abstracts in processing data. Therefore, this modified bibliometric analysis is able to put forward a comprehensive overview of research trends as well as identify gaps in the literature of the association between HIV-related stigma and contextual factors.

## 5. Conclusions

In conclusion, stigma and discrimination against patients with HIV/AIDS continues to be an urgent issue, given the epidemic’s dynamic nature in the extended life-long treatment. Future research and intervention will require a more intensive understanding of the impact of context as well as addressing the psycho-behavioral and social aspects of HIV care and treatment.

## Figures and Tables

**Table 1:** Type of data and analytical methods.

Type of Data	Unit of Analysis	Analytical Methods	Presentations of Results
Key words, Countries	Words	Frequency of co-occurrence	(1) Map of keywords clusters
Abstracts	Words	Exploratory factors analyses	(2) Top 50 constructed research domains (3) Clustering map of the landscapes constructed by these domains.
Abstracts	Papers	Latent Dirichlet Allocation	(4) 10 classifications of research topics
WoS classification of research areas	WoS research areas	Frequency of co-occurrence	(5) Dendrogram of research disciplines (WoS classification)

**Table 2:** Number of paper by study settings.

#	Country	Freq.	Percent	#	Country	Freq.	Percent	#	Country	Freq.	Percent
1	United States	406	14.26	42	Rwanda	10	0.35	84	Sri Lanka	3	0.11
2	South Africa	380	13.34	43	Spain	10	0.35	85	Tajikistan	3	0.11
3	China	230	8.08	44	New Zealand	9	0.32	86	Togo	3	0.11
4	India	177	6.21	45	Senegal	9	0.32	87	Trinidad and Tobago	3	0.11
5	Uganda	127	4.46	46	Taiwan	9	0.32	88	Turkey	3	0.11
6	Kenya	116	4.07	47	Burkina Faso	8	0.28	89	Albania	2	0.07
7	Canada	104	3.65	48	Georgia	8	0.28	90	Bolivia	2	0.07
8	Tanzania	82	2.88	49	Ukraine	8	0.28	91	Cuba	2	0.07
9	Australia	73	2.56	50	Argentina	7	0.25	92	Czech	2	0.07
10	Viet Nam	67	2.35	51	Chile	7	0.25	93	Gabon	2	0.07
11	Ethiopia	59	2.07	52	Guatemala	7	0.25	94	Gambia	2	0.07
12	Thailand	50	1.76	53	Italy	7	0.25	95	Hungary	2	0.07
13	Brazil	44	1.54	54	Lao	7	0.25	96	Kazakhstan	2	0.07
14	Ghana	44	1.54	55	Pakistan	7	0.25	97	Libya	2	0.07
15	Malawi	44	1.54	56	Bangladesh	6	0.21	98	Madagascar	2	0.07
16	Botswana	43	1.51	57	Barbados	6	0.21	99	Mali	2	0.07
17	United Kingdom	43	1.51	58	Colombia	6	0.21	100	Philippines	2	0.07
18	Zimbabwe	43	1.51	59	Greece	6	0.21	101	Suriname	2	0.07
19	Zambia	40	1.40	60	Sudan	6	0.21	102	Wallis and Futuna	2	0.07
20	Ireland	31	1.09	61	Belgium	5	0.18	103	Afghanistan	1	0.04
21	Japan	23	0.81	62	Estonia	5	0.18	104	Angola	1	0.04
22	Mexico	23	0.81	63	Germany	5	0.18	105	Benin	1	0.04
23	Indonesia	22	0.77	64	Singapore	5	0.18	106	Bulgaria	1	0.04
24	Peru	22	0.77	65	United Arab Emirates	5	0.18	107	Burundi	1	0.04
25	Hong Kong	21	0.74	66	Yemen	5	0.18	108	Central African Republic	1	0.04
26	Namibia	21	0.74	67	El Salvador	4	0.14	109	Cyprus	1	0.04
27	Puerto Rico	21	0.74	68	Myanmar	4	0.14	110	Denmark	1	0.04
28	Malaysia	20	0.70	69	Portugal	4	0.14	111	Fiji	1	0.04
29	Iran	19	0.67	70	Russian Federation	4	0.14	112	French Guiana	1	0.04
30	Nepal	18	0.63	71	Saudi Arabia	4	0.14	113	Grenada	1	0.04
31	Swaziland	16	0.56	72	Switzerland	4	0.14	114	Guinea	1	0.04
32	France	15	0.53	73	Croatia	3	0.11	115	Jersey	1	0.04
33	Mozambique	15	0.53	74	Egypt	3	0.11	116	Macedonia	1	0.04
34	Jamaica	14	0.49	75	Guyana	3	0.11	117	Micronesia	1	0.04
35	Cambodia	13	0.46	76	Honduras	3	0.11	118	Mongolia	1	0.04
36	Netherlands	13	0.46	77	Israel	3	0.11	119	Morocco	1	0.04
37	Cameroon	11	0.39	78	Jordan	3	0.11	120	Romania	1	0.04
38	Haiti	11	0.39	79	Kyrgyzstan	3	0.11	121	Solomon Islands	1	0.04
39	Sweden	11	0.39	80	Nicaragua	3	0.11	122	Somalia	1	0.04
40	Lebanon	10	0.35	81	Poland	3	0.11	123	Timor-Leste	1	0.04
41	Lesotho	10	0.35	82	Serbia	3	0.11	124	Tunisia	1	0.04
				83	Sierra Leone	3	0.11	125	Uruguay	1	0.04

**Table 3:** General characteristics of publications.

Year Published	Total # Papers	Total Citations	Mean Cite Rate Per Year	Total Usage Last 6 Month	Total Usage Last 5 Years	Mean Use Rate Last 6 Month	Mean Use Rate Last 5 Year
2017	633	806	1.27	1150	3455	1.82	1.09
2016	661	2418	1.83	712	5409	1.08	1.64
2015	632	4208	2.22	481	5930	0.76	1.88
2014	493	5691	2.89	363	5756	0.74	2.34
2013	520	8340	3.21	337	7136	0.65	2.74
2012	418	6992	2.79	172	4369	0.41	2.09
2011	356	6544	2.63	159	3068	0.45	1.72
2010	340	6818	2.51	131	2388	0.39	1.40
2009	286	7486	2.91	125	2133	0.44	1.49
2008	284	8846	3.11	127	1897	0.45	1.34
2007	243	7669	2.87	107	1521	0.44	1.25
2006	184	6523	2.95	58	1052	0.32	1.14
2005	146	6606	3.48	67	920	0.46	1.26
2004	98	4542	3.31	43	634	0.44	1.29
2003	81	7844	6.46	86	1099	1.06	2.71
2002	68	2874	2.64	25	284	0.37	0.84
2001	59	3175	3.17	18	334	0.31	1.13
2000	69	3491	2.81	28	395	0.41	1.14
1999	53	2396	2.38	12	226	0.23	0.85
1998	40	1021	1.28	11	123	0.28	0.62
1997	54	3866	3.41	14	213	0.26	0.79
1996	53	1704	1.46	9	159	0.17	0.60
1995	46	2460	2.33	12	189	0.26	0.82
1994	31	1776	2.39	8	132	0.26	0.85
1993	26	960	1.48	5	69	0.19	0.53
1992	24	1539	2.47	4	111	0.17	0.93
1991	12	565	1.74	2	24	0.17	0.40

**Table 4:** Top 50 research domains emerged from exploratory factor analysis of all abstracts’ contents.

No	Name	Keywords	Eigenvalue	Freq.	% Cases
1	Men (Msm; Sex Sexual)	msm; men; sex; sexual; risk	4.71	6175	50.94%
2	Care Providers	providers; provider; service; care; services; healthcare	1.61	5107	48.18%
3	Fsws; Female Sex	fsws; female; workers; clients; work; sex	1.75	3542	39.24%
4	Sexual Partners	partners; partner; sexual; disclosure; communication	1.28	3347	38.17%
5	Antiretroviral; Therapy	antiretroviral; therapy; adherence; medication; treatment; taking	2.63	4236	37.58%
6	Dna; Rt	dna; rt; viral; discrimination	1.92	2449	36.15%
7	Testing	testing; vct; test; counselling; uptake; tested; counseling; confidentiality	2.06	3227	31.50%
8	Reduction Intervention	intervention; trial; reduction; follow; control; reducing	2.27	2586	31.40%
9	Gender; Transgender	gender; transgender; women; identity	1.29	2412	31.23%
10	Depression and Anxiety; Psychological	anxiety; depression; psychological; distress; symptoms; mental; stress; psychosocial; physical	4.19	3187	28.76%
11	Children; Parents	children; parents; families; caregivers; family; affected	2.46	2544	27.82%
12	Gay Men	gay; bisexual; men; identity	1.42	2156	25.20%
13	Chronic Illness	illness; chronic; disease; conditions	2.48	1834	24.60%
14	Scale Scores; Factor	scale; factor; scores; developed; measures	1.35	1771	23.16%
15	General Population	general; population	1.48	1522	21.77%
16	Family Members	members; family; network	1.28	1466	19.45%
17	Coping Strategies	coping; internalized; strategies	1.18	1262	18.63%
18	Policy and Practice	practice; implications; policy	1.18	1300	18.55%
19	Substance Abuse	abuse; substance; alcohol; mental; violence	1.67	1517	18.37%
20	South Africa	africa; saharan; south	1.69	1745	17.06%
21	Transmission (PMTCT)	pmtct; mothers; transmission	1.63	1124	16.34%
22	QoL; Life Quality	qol; quality; life	1.5	1287	16.06%
23	Youth; Adults and Adolescents	youth; adolescents; young; adults	1.78	1181	15.74%
24	Income Countries	income; countries; global	3.09	1140	15.54%
25	Human Immunodeficiency	immunodeficiency; human	1.39	1460	14.97%
26	Behavior	behavior; behaviors	1.15	951	13.52%
27	Staff Training	staff; management; training	1.19	923	13.49%
28	Sexually Transmitted	transmitted; sexually; infections; sti	2.11	1384	12.77%
29	Drug Users	users; drug	1.45	968	12.63%
30	Black; States	black; states; american	1.25	805	11.75%
31	Majority	majority; respondents	1.27	753	11.66%
32	Cultural Beliefs	beliefs; cultural	1.24	750	11.33%
33	Association of Nurses	nurses; plwh; association	1.44	762	11.25%
34	Demographic Characteristics	characteristics; demographic	1.38	743	10.51%
35	Urban and Rural	rural; urban	1.31	751	10.46%
36	Counselling	counselling programme; programmes;	1.81	737	10.16%
37	Structural	structural	1.14	514	8.59%
38	Vaccine Trial	vaccine; trial; participation	1.38	567	8.19%
39	Order	order	1.25	421	7.04%
40	Common	common	1.16	420	7.02%
41	Prep	prep; exposure	1.54	365	5.06%
42	Resources	resources	1.22	296	4.95%
43	Behaviour	behaviour	1.13	289	4.83%
44	Tuberculosis (Tb	tb; tuberculosis	1.86	333	3.59%
45	Home	home	1.21	198	3.31%
46	India	india	1.23	195	3.26%
47	Screening	screening	1.56	195	3.26%
48	PLHIV	plhiv	1.18	154	2.57%
49	Hepatitis	hepatitis; hcv	1.98	213	2.52%
50	Sexual	couple; disclosure;	1.25	145	2.39%

**Table 5:** Most cited papers mentioning interventions.

#	Title	Journal	Citations	Years	Cite Rate
1	Interventions to reduce HIV/AIDs stigma: What have we learned?	Aids Education And Prevention	413	2003	27.5
2	AIDS-related Kaposi’s sarcoma: Prospective validation of the AIDS Clinical Trials Group staging classification	Journal Of Clinical Oncology	156	1997	7.4
3	A systematic review of interventions to reduce HIV-related stigma and discrimination from 2002 to 2013: how far have we come?	Journal Of The International Aids Society	136	2013	27.2
4	HIV Interventions to Reduce HIV/AIDS Stigma: A Systematic Review	Aids And Behavior	135	2011	19.3
5	Health work, female sex workers and HIV/AIDS: Global and local dimensions of stigma and deviance as barriers to effective interventions	Social Science & Medicine	95	2008	9.5
6	Willingness to volunteer in future preventive HIV vaccine trials: Issues and perspectives from three US communities	Journal Of Acquired Immune Deficiency Syndromes	84	2001	4.9
7	A Qualitative Study of the Barriers and Facilitators to Retention-in-Care Among HIV-Positive Women in the Rural Southeastern United States: Implications for Targeted Interventions	Aids Patient Care And STDs	77	2010	9.6
8	Getting me back on track”: The role of outreach interventions in engaging and retaining people living with HIV/AIDS in medical care	Aids Patient Care And STDs	72	2007	6.5
9	Linking sexual and reproductive health and HIV interventions: a systematic review	Journal Of The International Aids Society	70	2010	8.8
10	Global burden, distribution, and interventions for infectious diseases of poverty	Infectious Diseases Of Poverty	67	2014	16.8
11	Who Gets Tested for HIV in a South African Urban Township? Implications for Test and Treat and Gender-Based Prevention Interventions	Journal Of Acquired Immune Deficiency Syndromes	59	2011	8.4
12	Transgender stigma and health: A critical review of stigma determinants, mechanisms, and interventions	Social Science & Medicine	54	2015	18.0
13	Preventing discrimination against volunteers in prophylactic HIV vaccine trials: Lessons from a phase II trial	Journal Of Acquired Immune Deficiency Syndromes And Human Retrovirology	42	1998	2.1
14	Trial-related discrimination in HIV vaccine clinical trials	Aids Research And Human Retroviruses	40	2001	2.4
15	Informing Faith-Based HIV/AIDS Interventions: HIV-Related Knowledge and Stigmatizing Attitudes at Project FAITH Churches in South Carolina	Public Health Reports	33	2010	4.1
16	Eliminating the latent HIV reservoir by reactivation strategies Advancing to clinical trials	Human Vaccines & Immunotherapeutics	31	2013	6.2
17	It’s an Uphill Battle Everyday”: Intersectionality, Low-Income Black Heterosexual Men, and Implications for HIV Prevention Research and Interventions	Psychology Of Men & Masculinity	31	2013	6.2
18	A model for community representation and participation in HIV prevention trials among women who engage in transactional sex in Africa	Aids Care-Psychological And Socio-Medical Aspects Of Aids/HIV	27	2008	2.7
19	Uptake of prevention of mother to child transmission interventions in Kenya: health systems are more influential than stigma	Journal Of The International Aids Society	26	2011	3.7
20	Why blacks do not take part in HIV vaccine trials	Journal Of The National Medical Association	26	2007	2.4
21	HIV prevention interventions to reduce sexual risk for African Americans: The influence of community-level stigma and psychological processes	Social Science & Medicine	26	2014	6.5
22	Attitudes and beliefs related to HIV/AIDS in urban religious congregations: Barriers and opportunities for HIV-related interventions	Social Science & Medicine	25	2012	4.2
23	What can HIV vaccine trials teach us about future HIV vaccine dissemination?	Vaccine	25	2008	2.5
24	Barriers to participation in HIV drug trials: a systematic review	Lancet Infectious Diseases	22	2006	1.8
25	Interventions to Improve Psychological Functioning and Health Outcomes of HIV-Infected Individuals with a History of Trauma or PTSD	Current HIV/Aids Reports	22	2012	3.7
26	Interventions to reduce the sexual risk behaviour of injecting drug users	International Journal Of Drug Policy	21	2005	1.6
27	Articulating A Rights-Based Approach to HIV Treatment and Prevention Interventions	Current HIV Research	21	2011	3.0
28	Experiences in conducting multiple community-based HIV prevention trials among women in KwaZulu-Natal, South Africa	Aids Research And Therapy	21	2010	2.6
29	Willingness to Participate in HIV Vaccine Trials among Men Who Have Sex with Men in Chennai and Mumbai, India: A Social Ecological Approach	Plos One	20	2012	3.3
30	Community empowerment and involvement of female sex workers in targeted sexual and reproductive health interventions in Africa: a systematic review	Globalization And Health	19	2014	4.8
31	Why Culture Matters in Health Interventions: Lessons From HIV/AIDS Stigma and NCDs	Health Education & Behavior	18	2014	4.5
32	Willingness of Chinese injection drug users to participate in HIV vaccine trials	Vaccine	18	2008	1.8
33	What HIV-Positive MSM Want from Sexual Risk Reduction Interventions: Findings from a Qualitative Study	Aids And Behavior	18	2012	3.0
34	Perceptions of barriers and facilitators to participation in clinical trials in HIV-Positive latinas: A pilot study	Journal Of Womens Health	16	2007	1.5
35	Community-based HIV/AIDS interventions to promote psychosocial well-being among people living with HIV/AIDS: a literature review	Health Psychology And Behavioral Medicine	15	2013	3.0
36	Limited role of culture conversion for decision-making in individual patient care and for advancing novel regimens to confirmatory clinical trials	BMC Medicine	15	2016	7.5
37	Preparedness for AIDS vaccine trials in India	Indian Journal Of Medical Research	15	2008	1.5
38	Community preparedness for HIV vaccine trials in the Democratic Republic of Congo	Culture Health & Sexuality	14	2006	1.2
39	Community-based HIV prevention interventions that combat anti-gay stigma for men who have sex with men and for transgender women	Journal Of Public Health Policy	14	2013	2.8
40	Perceptions of a community sample about participation in future HIV vaccine trials in South India	Aids And Behavior	14	2007	1.3
41	Systematic review of stigma reducing interventions for African/Black diasporic women	Journal Of The International Aids Society	14	2015	4.7
42	‘Rumours’ and clinical trials: a retrospective examination of a pediatric malnutrition study in Zambia, southern Africa	Bmc Public Health	14	2010	1.8
43	Gender-Specific HIV Prevention Interventions for Women Who Use Alcohol and Other Drugs: The Evolution of the Science and Future Directions	Jaids-Journal Of Acquired Immune Deficiency Syndromes	12	2015	4.0
44	The influence of social determinants on evidence-based behavioral interventions-considerations for implementation in community settings	Translational Behavioral Medicine	11	2012	1.8
45	Women, reproductive rights, and HIV/AIDS: Issues on which research and interventions are still needed	Journal Of Health Population And Nutrition	11	2006	0.9
46	What Interventions Are Needed for Women and Girls Who Use Drugs? A Global Perspective	Journal Of Acquired Immune Deficiency Syndromes	10	2015	3.3
47	The preexposure prophylaxis revolution; from clinical trials to programmatic implementation	Current Opinion In HIV And Aids	10	2016	5.0
48	Antiretroviral interventions to reduce mother-to-child transmission of human immunodeficiency virus: challenges for health systems, communities and society	Bulletin Of The World Health Organization	10	2000	0.6
49	If It’s Not Working, Why Would They Be Testing It?”: mental models of HIV vaccine trials and preventive misconception among men who have sex with men in India	BMC Public Health	9	2013	1.8
50	Effectiveness of Sport-Based HIV Prevention Interventions: A Systematic Review of the Evidence	Aids And Behavior	9	2013	1.8
